# After many a summer dies the swan

**DOI:** 10.1093/nsr/nwz046

**Published:** 2019-05-24

**Authors:** Chung-I Wu

**Affiliations:** 1 School of Life Science, Sun Yat-Sen University, China; 2 Department of Ecology and Evolution, University of Chicago, USA; 3 Section Editor for Life Sciences at NSR

Just how different are humans from other hominids at the genic level? How many gene replacements are necessary to transform a chimpanzee into something that, in a subway train, would not cause massive panic (perhaps only a bit of intrigue)? Genome sequencing has revealed more than 3 million bp differences but those differences offer no insight into the functional questions answerable only by gene replacement.

In this issue, Shi *et al.* provide the experimental support for the neoteny hypothesis, by which juvenile development in humans is posited to have been extended into adulthood [1]. In this view, human adults are akin to sexually mature babies. On the flip side, Aldous Huxley, in his science fiction *After Many a Summer Dies the Swan*, depicts an immortal human, the Fifth Earl of Gonister, who lived to 200 and became an ape, presumably after prolonged development [2].

Although numerous differences in morphological, reproductive and behavioral traits exist between humans and apes, all these traits might be correlated. Louis Bolk, a Dutch anatomist, characterized these differences as being juvenile-prone in humans [3]. The most notable ones are the size and bulbous shape of the cranium of adult humans that resemble the fetal features.

According to this neoteny hypothesis, the bulk of biological differences between human and other apes could be driven by a small number of genes controlling the rate of embryonic and fetal development. Shi *et al.* transduce the human MCPH1 gene into the rhesus monkey. Prior extensive studies of this gene in mouse and monkey have implicated MCPH1 in brain development. What is remarkable is that the transgenic monkeys show delayed neuronal maturation, human-like delayed expressions of neuron differentiation genes and improved memory/reaction time. It thus appears that a single gene could have some detectable effects that hint at the neotenic development of the brain.

Studies such as those by Shi *et al.* and others [4,5] on monkey cloning may foreshadow what will come in the near future. The evolution of human intelligence could be understood even before we unravel the intricate mechanistic details of the human brain.
